# Evaluation of Antimicrobial Drug Use and Concurrent Infections During Hospitalization of Patients With COVID-19 in Japan

**DOI:** 10.1001/jamanetworkopen.2022.0040

**Published:** 2022-02-18

**Authors:** Junpei Komagamine, Taku Yabuki, Kohei Matsumoto, Nao Tanaka

**Affiliations:** 1 Department of Internal Medicine, National Hospital Organization Tochigi Medical Center, Nakatomatsuri, Utsunomiya, Tochigi, Japan

## Abstract

This cross-sectional study examines the prevalence and necessity of antimicrobial drug use among patients who are hospitalized with COVID-19 infection in Japan.

## Introduction

A recent meta-analysis^1,2^ and a prospective study^3^ reported that bacterial coinfection and secondary infection among patients with COVID-19 infection are uncommon. However, in these studies, antimicrobial drugs were prescribed for approximately 70% of patients with the disease.^2^ Therefore, data are needed on patients with COVID-19 infection who do not routinely receive an antimicrobial drug prescription, to identify the true rate of concurrent infection in this patient population. At the National Hospital Organization Tochigi Medical Center in Utsunomiya, Tochigi, Japan, since the beginning of the COVID-19 pandemic, antimicrobial drugs have not been prescribed for patients with COVID-19 infection unless their symptoms are suggestive of another infectious disease. Therefore, we investigated the prevalence of antimicrobial drug use and concurrent infections among patients with COVID-19 during hospitalization.

## Methods

This retrospective cross-sectional study obtained and analyzed data from electronic medical records. The institutional ethics committee of the National Hospital Organization Tochigi Medical Center approved this study and formally waived the informed consent requirement because deidentified data were collected without contacting the patients. We followed the Strengthening the Reporting of Observational Studies in Epidemiology (STROBE) reporting guideline.

All symptomatic patients with laboratory-confirmed COVID-19 infection who were hospitalized at the National Hospital Organization Tochigi Medical Center from November 1, 2020, to October 9, 2021, were eligible. Excluding 47 patients who were transferred to other hospitals for intensive care, the final analysis included 1056 patients (eMethods in the Supplement). At the hospital, patients with COVID-19 infection were treated according to World Health Organization guidelines.^4^ The COVID-19 care team included 2 physicians on the antimicrobial stewardship team.

Information on demographic characteristics, microbial test results, and medication use during hospitalization was extracted from electronic medical records. The severity of COVID-19 was assessed using the World Health Organization classification.^5^ The primary outcomes were the prevalence of antimicrobial drug use and the proportion of patients with microbiologically confirmed concurrent infections during hospitalization. Descriptive statistics were used to report patient characteristics and outcomes. We used Stata, version 15 (LightStone).

## Results

Of the 1056 patients analyzed (median [IQR] age, 50 [36-61] years; 669 men [63.4%] and 387 women [36.7%]) (Table 1), 533 (50.5%) had mild, 313 (29.6%) had moderate, 203 (19.2%) had severe, and 7 (0.7%) had critical COVID-19 infection. During hospitalization, 9 patients (0.9%) died, 1046 patients (99.1%) recovered, and 1 patient was transferred to another hospital before recovery for social reasons. Although antimicrobial drugs were prescribed for 104 patients (9.9%) before admission, only 18 patients (1.7%) received any antimicrobial drugs during hospitalization (Table 2). Of these 18 patients, 15 used the drug as treatment, and 3 used it as a prophylaxis. Seven microbiologically confirmed infectious diseases other than COVID-19 were detected in 6 patients (0.6%) during hospitalization. Even if mild COVID-19 cases were excluded, they occurred in only 5 patients (0.9%).

**Table 1.** Clinical and Demographic Characteristics of Hospitalized Symptomatic Patients With COVID-19

Characteristic	Patients, No. (%) (N = 1056)
Age, median (IQR), y	50 (36-61)
Men	669 (63.4)
Women	387 (36.7)
Race and ethnicity^a^	
East Asian	976 (92.4)
Southeast Asian	41 (3.9)
South Asian	26 (2.5)
Unknown^b^	13 (1.2)
BMI, median (IQR)	24.1 (21.5-27.0)
Vaccination status for COVID-19	
None	969 (91.7)
Once	64 (6.1)
Twice	23 (2.2)
Current smoking	274 (26.0)
Medical history	
Ischemic heart disease	16 (1.5)
Diabetes	123 (11.7)
Dialysis	0
COPD or asthma	83 (7.9)
Active cancer	9 (0.9)
Immunosuppression drug use	15 (1.4)
No. of days to admission from symptom onset, median (IQR), d	5 (3-7)
Fever at admission	643 (60.9)
Presence of pneumonia^c^	514 (48.7)
Treatment	
Remdesivir	0
Dexamethasone	200 (18.9)
Tocilizumab	16 (1.5)
Casirivimab and imdevimab	82 (7.8)
Duration of hospital stay, median (IQR), d	6 (4-8)

Abbreviations: BMI, body mass index (calculated as weight in kilograms divided by height in meters squared); COPD, chronic obstructive pulmonary disease.

^a^ Race and ethnicity were self-reported.

^b^ Data were missing.

^c^ Defined as clinical symptoms and crackles on physical examination or as infiltration on chest imaging.

**Table 2.** Prevalence of Antimicrobial Drug Use and Concurrent Infectious Diseases Among Patients With COVID-19, by Severity^a^

Status	Patients by COVID-19 severity, No. (%)				
	Total (N = 1056)	Critical (n = 7)	Severe (n = 203)	Moderate (n = 313)	Mild (n = 533)
No antimicrobial drugs from symptom onset until discharge	936 (88.4)	5 (71.4)	170 (83.7)	282 (90.1)	479 (89.9)
Prehospital antimicrobial drug use					
Any use	104 (9.9)	1 (14.3)	21 (10.3)	30 (9.6)	52 (9.8)
Newly prescribed for symptoms	99 (9.4)	0	21 (10.3)	28 (9.0)	50 (9.4)
Long-term use^b^	5 (0.5)	1 (14.3)	0	2 (0.6)	2 (0.4)
Antimicrobial drug use during hospitalization					
Any use	18 (1.7)	2 (28.6)	12 (5.9)	2 (0.6)	2 (0.6)
Newly prescribed	16 (1.5)	1 (14.3)	12 (5.9)	1 (0.6)	2 (0.6)
Long-term use^b^	2 (0.2)	1 (14.3)	0	1 (0.6)	0
Concurrent infectious diseases during hospitalization					
Clinically diagnosed	13 (1.2)	1 (14.3)	10 (4.9)	0	2 (0.4)
Microbiologically confirmed	6 (0.6)	1 (14.3)	4 (2.0)	0	1 (0.2)

^a^ Severity was assessed when the status of the patient was the worst. Severity was defined according to World Health Organization guidelines.

^b^ Two patients were using trimethoprim sulfamethoxazole for pneumocystis pneumonia prophylaxis, and 3 patients were using clarithromycin for asthma or chronic obstructive pulmonary disease. Because there were no indications for clarithromycin in the 3 patients with asthma or chronic obstructive pulmonary disease, it was stopped after hospital admission.

## Discussion

The findings revealed that concurrent infectious diseases rarely occurred despite the infrequent use of antimicrobial drugs during hospitalization among patients with COVID-19 infection, most of whom had noncritical cases. This finding supports the results of recent studies showing that concurrent bacterial infections were uncommon in patients with COVID-19 infection.^1,2,3^ Given that most patients with noncritical severity recovered without antimicrobial drugs, the use of most antimicrobial drugs to treat noncritical cases in many hospitals might be unnecessary. Antimicrobial drugs should be used cautiously to treat patients with COVID-19 infection.

The strengths and importance of this study were the assessment of the use of all antimicrobial drugs (regardless of the reason for use) and the finding of an extremely low rate of antimicrobial drug use compared with that in previous studies.^2^ The limitations were the retrospective data collection, single-center design, short-term follow-up, and exclusion of patients who were transferred to other hospitals for intensive care. Moreover, a positive result on a microbial test did not necessarily indicate true infection. In addition, a few patients who had active cancer or were immunocompromised were included.

## Notes

Supplement.
eMethods.
