# Supplementary material for: Evaluation of Antimicrobial Drug Use and Concurrent Infections During Hospitalization of Patients With COVID-19 in Japan
Source: JAMA Netw Open. 2022 Feb 18;5(2):e220040. doi: 10.1001/jamanetworkopen.2022.0040 (PMC8857682; doi:10.1001/jamanetworkopen.2022.0040)
Supplement: Supplement. — eMethods. [file jamanetwopen-e220040-s001.pdf]

Supplementary Online Content

Komagamine J, Yabuki T, Matsumoto K, Tanaka N. Evaluation of antimicrobial drug use and concurrent infections during hospitalization of patients with COVID-19 in Japan. *JAMA Netw Open*. 2022;5(2):e220040. doi:10.1001/jamanetworkopen.2022.0040

eMethods.

This supplementary material has been provided by the authors to give readers additional information about their work.

© 2022 Komagamine J et al. *JAMA Network Open.*

eMethods

Settings: The National Hospital Organization Tochigi Medical Center is a 350-bed community acute care hospital, and it is one of the two largest hospitals providing care for approximately 0.5 million people in Utsunomiya City in Tochigi Prefecture (Japan). Since the beginning of the COVID-19 pandemic, the National Hospital Organization Tochigi Medical Center has provided care for approximately one-fifth of all COVID-19 patients in Utsunomiya. The National Hospital Organization Tochigi Medical Center has no intensive care units but high acuity units.

COVID-19 care in this region (Utsunomiya): At the beginning of the COVID-19 pandemic, the National Hospital Organization Tochigi Medical Center was the only hospital designated for COVID-19 care in Utsunomiya, Tochigi, Japan. All COVID-19 patients, including asymptomatic COVID-19 patients, in Utsunomiya were hospitalized in this hospital. However, care for critical COVID-19 was provided by two university hospitals in other parts of Tochigi Prefecture and one tertiary care hospital in Utsunomiya. As of December 19, 2021, eight community care hospitals provide care for noncritical COVID-19. If intensive care is judged to be needed, and the patient prefers it, COVID-19 patients are transferred from these eight hospitals to the two university hospitals and one tertiary care hospital. The policy to admit all COVID-19 patients without specific reasons in this region has not changed since the beginning of the COVID-19 pandemic. Among patients with noncritical COVID-19, patients with a higher severity of COVID-19 or higher risk factors associated with progression to critical COVID-19 are admitted to the National Hospital Organization Tochigi Medical Center.

Inclusion and exclusion criteria: All symptomatic COVID-19 patients who had received confirmation by SARS-CoV-2 nucleic acid or antigen testing were included. Only patients who were hospitalized in our hospital during the study period (from November 1, 2020, to October 9, 2021) were included. Asymptomatic COVID-19 patients (n=39) and clinically diagnosed COVID-19 patients without laboratory confirmations (n=5) were excluded.

© 2022 Komagamine J et al. *JAMA Network Open.*
